# On the Pharmacopœia

**Published:** 1804-06-01

**Authors:** 


### *To the Editors of the Medical and Physical Journal.*

GENTLEMEN,

TEMPERATE discussion is always serviceable to science. In this point of view, Mr. Hume's Remarks on the Edinburgh Pharmacopœia, published in your last Number, are highly valuable. In this city, where the students are by no means disposed blindly to accept the doctrines of their teachers, or to esteem a work the more for being the production of a College, similar observations have been frequently made, and as frequently been replied to. Every thing connected with pharmacy is particularly interesting to me; I therefore have paid a good deal of attention tion to these discussions; and upon the whole, it appears to me, that the objections which have been made, have generally been the consequence of partial views, inattention, or ignorance. But as Mr. Hume's very candid Observations are the first which have appeared in a publication, which will also admit a candid Reply, I shall take the liberty of answering them, principally with the views of exciting scientific discussion, and obtaining information.

Every one will regret with Mr. Hume, that so many differences, in every particular, exist between the Pharmacopœias of London and Edinburgh; but I do not understand how, upon this regret, he should engraft a general charge of want of precision and simplicity against either or both the Colleges. The charge to many will appear unfounded, and should not have been made without reasons to support it.

Much certainly remains to be done, before a Pharmacopœia shall be composed free from errors and numerous ambiguities; but Mr. Hume has been guilty of illiberality, in applying the epithet disgraceful to these errors and ambiguities, unless he is able either to point out how they may be avoided, or, what would be preferable, and undoubtedly the most advantageous employment both of Mr. Hume's time and abilities, to publish one himself, free from abiguity and error; his improvements could not fail to be acceptable to both Colleges.

Mr. Hume, however, seems to be diffident of his own abilities, and to despair of seeing perfection as well as uniformity in the Pharmacopœias, unless a deputation from each College be authorized to execute this important work. By such a measure, we might certainly procure an Imperial Pharmacopœia, in place of the London and Edinburgh Pharmacopæias; but as certainly, it would be less perfect than either. There is an old vulgar adage, "Many cooks spoil the broth;" and so would many Doctors the prescription. But I find that Critics can disagree as well as Doctors; and on this occasion shall screen myself by pitting one against another, and referring Mr. Hume to the concluding paragraph of the Edinburgh Review of the Edinburgh Pharmacopœia, vol. iii. p. 467. If Mr. Hume, after reading it, shall persist in his opinion, it will be incumbent on him to prove the practicability of his plan.— In attempting to do this, he may perhaps find some assistance in the Preface to Van Mon's Pharmacopœia, where he proposes, that at the peace there should be a convention of deputies from all the states of Europe, to establish an uniformity of pharmaceutical practice over this quarter of the globe at least.

Mr. Hume now proceeds to his specific charges against the Edinburgh College. The first is, that we find the same name sometimes put adjectively and sometimes substantively, without any apparent intention; on the contrary, for obvious reasons, liable to lead into error. Now, his obvious reasons are no more apparent to me, than the intention of the College is to him; I must therefore request him to explain them. "The adjective, compositum, is applied at random in all its cases." Mr. Hume surely does not mean that the different cases of compositum are used at random! "Why tack it," says he, "to the tinct. cassiæ sennæ, and not to tinct. mimosæ catechu?" Because, in the latter case, catechu is the only active ingredient; and in the former, cassia is not the only active ingredient. Again, "Why tack it to pil. rhei; there *is* no other Formula with which these may be confounded?" There is not in the Edinburgh Pharmacopœia; but these pills are *compound*, and any other College, or any Hospital or Individual, may publish a Formula for simple rhubarb pills.

To all the objections to the introduction of the Linnæan and chemical names, there is this obvious answer, that Pharmacy is but the application of Natural History and Chemistry to a particular purpose, and cannot deviate from the language adopted in these sciences, without manifest impropriety. The verbosity, the great objection, I have no doubt will be gradually corrected by means of systematic abreviations. In all probability, Linnæus never ventured even to hope that his name would ever be introduced into Pharmacy; but I think that such a prospect would have afforded him the highest gratification. The remaining part of the sentence, "any farther than in Systems of Botany, Natural History, and in a few other similar productions, these names seem inadmissible," is, to me, unintelligible. The objection to the chemical names, that we might as well, in geographical description, substitute solution of muriat of soda, &c. for the word ocean, or oxyd of hydrogen for water, is frivolous. Oxyd of hydrogen has not been adopted by chemists for water, and geography is not a branch of chemistry.

Mr. Hume seems to be surprised at the title, emplastrum gummosum. What would he have it called? Mr. Hume tells us, that the conjunction et and the preposition cum, are are often used very ambiguously; that in some instances they mean chemical combination, and in others mere mechanical mixture. The cum might, in general, have been avoided; but Mr. Hume's objection of inconsistency would only be relevant, if the College had pretended to use these words in any other sense than as mere copulatives. His objection to sulphas potassæ cum sulphure seems valid, but I much doubt that it is a sulphite of potass, for I have never been able to disengage sulphureous acid from it by means of sulphuric acid. The process and the substance itself have been retained, I have no doubt, in compliance with experience and prejudice. To Mr. Hume's questions respecting the oxidum antimonii cum phosphate calcis, the following answer may be given. We have no better or more explicit title. Pulvis algarothi mixed with phosphat of lime will not produce the pulvis antimonialis of the London College, because, although Mr. Hume apprehends it to be the most *uniform* oxyd of antimony, it is a submuriat and not an oxyd. That the Edinburgh title expresses the real constitution of the substance in question was proved by Mr. Chenevix. Flavus is perhaps an unnecessary epithet to the sub-sulphat of mercury. But in the substitute proposed for the whole title by Mr. Hume, he has given us a bad specimen of his skill in contriving unobjectionable names. Hydrarg. flavus would mean metallic quicksilver of a yellow colour. I totally differ from Mr. Hume in thinking that sub and super are unnecessary expletives; on the contrary, I think them a most material improvement, and from a subsequent criticism have some idea that Mr. Hume does not understand their application in chemical nomenclature. With regard to the comparative strength of crude and purified opium, I again disagree from Mr. Hume, for I consider all the processes used for *purifying* opium as processes for *spoiling* it. Mr. Hume tells us, that cerussa chiefly consists of carbonas plumbi; I very much doubt it. What he means by there being too much *play* on the word oxidum, I do not know; it is evidently much used, and with great propriety. The word *impurum* is used for the sake of truth, because the articles to which it is affixed are *impure*; and impure articles are admitted, because they are sufficient for the purpose, and because apothecaries, however they might charge their patients, would not be at the expence of procuring them pure. That there are many varieties of calamine, so far from being an objection is an additional reason for the use of the epithet Impurum. It is hardly possible to free sulphat sulphat of zinc entirely from iron; but its presence is not injurious, and the acid is added because the sulphat of zinc employed by apothecaries contains oxyd of zinc in excess. With regard to the presence of sulphuric acid in the solutio acetitis zinci, Mr. H. is probably right; but the mode of preparing it is not only the most convenient, but the best. The reason, I apprehend, for retaining the two carbonats of iron is, that apothecaries will in general procure it from the trading chemists, while directions, by which it may be prepared in a few hours, may often be useful. I apprehend that no carbonic acid remains in the ammoniaretum cupri; what proof has Mr. H. to the contrary? Mr. Hume asks, if sub be properly added to acetis cupri, why is it not to acetis plumbi? I answer, simply, because the former is a sub-acetite and the latter is not.

Such, Gentlemen, are the arguments by which I have heard similar criticisms refuted when they have been the subject of discussion among my fellow students; and if they are not always satisfactory, they will at least show that much may be said on both sides. On the new Pharmacopœia, both with regard to its nomenclature and its substance, some observations worthy of notice will be found in the Review already mentioned; in Duncan's, jun. New Dispensatory; and in Murray's Elements of Pharmacy.

A STUDENT OF PHARMACY.

